# PedCAPNETZ – prospective observational study on community acquired pneumonia in children and adolescents

**DOI:** 10.1186/s12890-019-1013-5

**Published:** 2019-12-09

**Authors:** Martin Wetzke, Matthias Volkmar Kopp, Jürgen Seidenberg, Christian Vogelberg, Tobias Ankermann, Christine Happle, Gesche Voigt, Holger Köster, Thomas Illig, Christiane Lex, Antje Schuster, Marcus Panning, Grit Barten, Gernot Rohde, Tobias Welte, Gesine Hansen, pedCAPNETZ Study Group

**Affiliations:** 1 Department of Pediatric Pneumology, Allergology and Neonatology, Hannover Medical School, Carl-Neuberg-Straße 1, D -, 30625 Hannover, Germany; 2 German Center for Infection Research (DZIF), Site Hannover-, Braunschweig, Germany; 3 German Center for Lung Research DZL, Airway Center North (ARCN) Lübeck and Biomedical Research in End-stage and Obstructive Lung Disease Hannover (BREATH), Hannover, Germany; 4 Department of Pediatric Allergy and Pulmonology, Clinic of Pediatrics UKSH, University of Luebeck, Luebeck, Germany; 5 Department of Pediatric Pneumology and Allergology, Universitätsklinik für Kinder- und Jugendmedizin Oldenburg, Oldenburg, Germany; 6 University Children’s Hospital, Technical University Dresden, Dresden, Germany; 7 Department of Pediatric Pulmonology, Clinic of Pediatrics UKSH, University of Kiel, Kiel, Germany; 8 Hannover Unified Biobank, Hannover Medical School, Hannover, Germany; 9 Department of Pediatric Pulmonology, University of Göttingen, Göttingen, Germany; 10 Department of Pediatric Pulmonology, University of Düsseldorf, Düsseldorf, Germany; 11 Institute of Virology, University of Freiburg, Freiburg, Germany; 12 CAPNETZ Foundation, Hannover, Germany; 13 Department of Respiratory Medicine, University Hospital Frankfurt, Frankfurt, Germany; 14 Department of Pulmonary Medicine, German Centre for Lung Research, Hannover Medical School, Hannover, Germany; 15 Cluster of Excellence RESIST (EXC 2155), Hannover Medical School, Carl-Neuberg-Str. 1, 30625 Hannover, Germany

## Abstract

**Background:**

Pediatric community acquired pneumonia (pedCAP) is one of the leading causes for childhood morbidity accounting for up to 20% of pediatric hospital admissions in high income countries. In spite of its high morbidity, updated epidemiological and pathogen data after introduction of preventive vaccination and novel pathogen screening strategies are limited. Moreover, there is a need for validated recommendations on diagnostic and treatment regimens in pedCAP. Through collection of patient data and analysis of pathogen and host factors in a large sample of unselected pedCAP patients in Germany, we aim to address and substantially improve this situation.

**Methods:**

pedCAPNETZ is an observational, multi-center study on pedCAP. Thus far, nine study centers in hospitals, outpatient clinics and practices have been initiated and more than 400 patients with radiologically confirmed pneumonia have been enrolled, aiming at a total of 1000 study participants. Employing an online data base, information on disease course, treatment as well as demographical and socioeconomical data is recorded. Patients are followed up until day 90 after enrollment; Comprehensive biosample collection and a central pedCAPNETZ biobank allow for in-depth analyses of pathogen and host factors. Standardized workflows to assure sample logistics and data management in more than fifteen future study centers have been established.

**Discussion:**

Through comprehensive epidemiological, clinical and biological analyses, pedCAPNETZ fills an important gap in pediatric and infection research. To secure dissemination of the registry, we will raise clinical and scientific awareness at all levels. We aim at participating in decision making processes for guidelines and prevention strategies. Ultimately, we hope the results of the pedCAPNETZ registry will help to improve care and quality of life in pedCAP patients in the future.

## Background

Community acquired pneumonia (CAP) is one of the leading global causes for childhood morbidity (1). Although morbidity and mortality due to pneumonia in adult patients have been reduced dramatically over the past decade, pediatric CAP (pedCAP) is still associated with high mortality and accounts for up to 20% of pediatric hospital admissions also in high income countries (1). Globally, pedCAP is associated with significant health care costs and impacts quality of life in many patients and their caregivers (2). In Germany, it is estimated that the cost for children hospitalized with pedCAP reaches more than 200 Million Euro per year (3). The annual numbers of German hospitalized pedCAP patients in the years 2006 to 2016 illustrate an incidence of around 40 cases per 10.000 children below the age of fifteen years and around 90 cases per 10.000 children below the age of five years (3). Although this number is just a rough estimate given the mode of data collection based on administrative data and ICD-10 coding without proper epidemiological study design, it is similar to estimates from the USA, where the overall hospitalization rate due to CAP was to be around 16 cases/10.000 in all children and adolescents, with the highest hospitalization rate in children below the age of two years (62/10.000 children) (4).

In spite of its high prevalence, critical gaps in our knowledge on pedCAP exist. For Germany, most data on pedCAP was collected in studies with small, monocentric cohorts that were recruited before implementation of the pneumococcal conjugate vaccine, lacking stringent clinical or radiographic inclusion criteria (5). Although the majority of pedCAP patients is treated in an outpatient manner, almost all recent studies conducted thus far focused on severe, hospitalized pedCAP (5). As only every fourth to fifth CAP case in Western Europe is hospitalized, these studies does by no means reflect the full spectrum of clinical pedCAP reality (6).

Besides the lack of updated epidemiological data, there is also no recent data on optimized age dependent diagnostic and therapeutic regimens in pedCAP. While in adults, well-established CAP risk stratification scores based on the implementation of clinical and laboratory markers facilitate decision making, e.g. with regard to antibiotic treatment and hospitalization, this is not the case for pedCAP (7–10). In 2011, the American Pediatric Infectious Diseases Society extrapolated severity criteria from the adult American CAP guideline for pediatric use, but the value of this scoring was shown to be very poor in clinical reality (11, 12). Only recently, a model to estimate the risk for moderate to severe pneumonia outcome in children based on ten to twenty patient, laboratory, and radiographic characteristics has been proposed, but independent studies to illustrate the true value of this scoring are pending (13).

Although highly desirable, clear clinical criteria and validated recommendations on how to limit the use of antibiotics in ambulatory pedCAP care are missing. For example, current recommendations for pedCAP management are mainly based on study data from the 1980ies and 1990ies, and the evidence level of the majority of these recommendations is low (14). As current diagnostic tools lack accuracy and are unable to delineate different disease subtypes such as viral vs. bacterial pedCAP, up until today, the diagnosis of pedCAP is mainly based on simple diagnostic criteria such as tachypnea and fever (15). The majority of guidelines suggest antibiotic treatment in all children with severe pedCAP (Additional file 1: Table S1), with different recommendations regarding the routine use of antibiotics in other, highly prevalent patient groups such as infants and toddlers with milder symptoms.

Several biomarkers have been suggested to assess the severity and etiology of CAP (16). Only few novel studies on biomarkers in viral vs. bacterial pedCAP exist C-reactive protein (CRP), white cell counts (WBC) and absolute neutrophil number in combination with fever, oxygen saturation, fluid uptake and absence of rhinorrhea still remain the most promising “biomarkers” to predict bacterial pedCAP (17). Other potential biomarkers that are helpful in clinical decision making such as interleukin 6/interleukin 10 ratio or lipocalin-2 and syndecan-4 levels in the serum have either only been tested in adult CAP patients or shown to have no added value compared to CRP and WBC count and differentiation in ped CAP (18, 19).

Furthermore, national data on current pathogen spectra in German pedCAP cases is lacking. Widespread implementation of the pneumococcal and *Haemophilus influenzae* type b (Hib) conjugate vaccines significantly reduced the incidence of pneumonia and hospitalization in children associated with these pathogens (20–22). But after the change in vaccination protocols the pathogen spectrum in pedCAP appears to have significantly shifted towards a virus dominated etiology: In large cohorts of hospitalized pediatric pneumonia patients, respiratory syncytial virus (RSV), rhinovirus (RV), and human metapneumovirus were the most frequently detected viral pathogens (23, 24). The increase in detection of viral strains in pedCAP may be caused by a shift in disease causing pathogens after vaccination, but could also be due to improved detection strategies. The increased sensitivity and availability of detection methods such polymerase chain reaction (PCR) has significantly facilitated detection of viruses in biosamples of patients with acute respiratory infections (25). However, the clinical significance of detections of viral strains in patients with pneumonia is often unclear. In a recent case control study in pedCAP cases below the age of five years and matched healthy controls, nasopharyngeal detection of influenza, metapneumovirus and RSV was significantly associated with pedCAP, but RV appeared to be not more frequent in pedCAP cases as compared to controls (24).

Taken together, recent epidemiological, clinical, biomarker, and pathogen data on pedCAP is scarce. By contrast, a plethora of data and regularly updated guidelines for the management of adult CAP exist. In this regard, major contributions were made by the German competence network for CAP (CAPNETZ) (6). The underlying adult CAPNETZ cohort with more than 10.000 recruited patients represents one of the largest and best characterized pneumonia cohorts worldwide, and current German guidelines on CAP management in adults were mainly based on data from this study (26). Previously, CAPNETZ focused exclusively on adult CAP cases and did not recruit pediatric or adolescent pneumonia patients. The newly formed pedCAPNETZ initiative aims to complement the adult CAPNETZ study and has set the goal to gather comprehensive data on pedCAP in Germany.

## Methods/design

### Aims

Similar to the aims defined during the initial setup of the adult CAPNETZ registry (6), pedCAPNETZ wants to provide the structure to fundamentally improve the knowledge on epidemiology, etiology and management of community acquired pneumonia in children and adolescents. The pedCAPNETZ study aims to provide current and in-depth clinical and molecular data on pedCAP in Germany.

The specific aims of pedCAPNETZ are:

‐to describe the full spectrum of pedCAP severity in Germany by capturing data from mild to severely affected pedCAP cases,

‐to assess current diagnostic and therapeutic approaches and their efficacy in pedCAP management,

‐to develop clinical scores for early risk stratification for severe pedCAP,

‐to identify and validate novel clinical or biological markers in pedCAP,

‐to analyze pedCAP pathogen spectra via comprehensive microbiome/virome and resistance screenings,

‐to raise clinical and scientific awareness of pedCAP at all levels and be open to a wide range of collaborations and interventional studies,

‐to participate in decision making processes for guidelines and prevention strategies to ultimately improve care and quality of life in pedCAP patients in the future.

### Study design and assessments

**Fig. 1:**
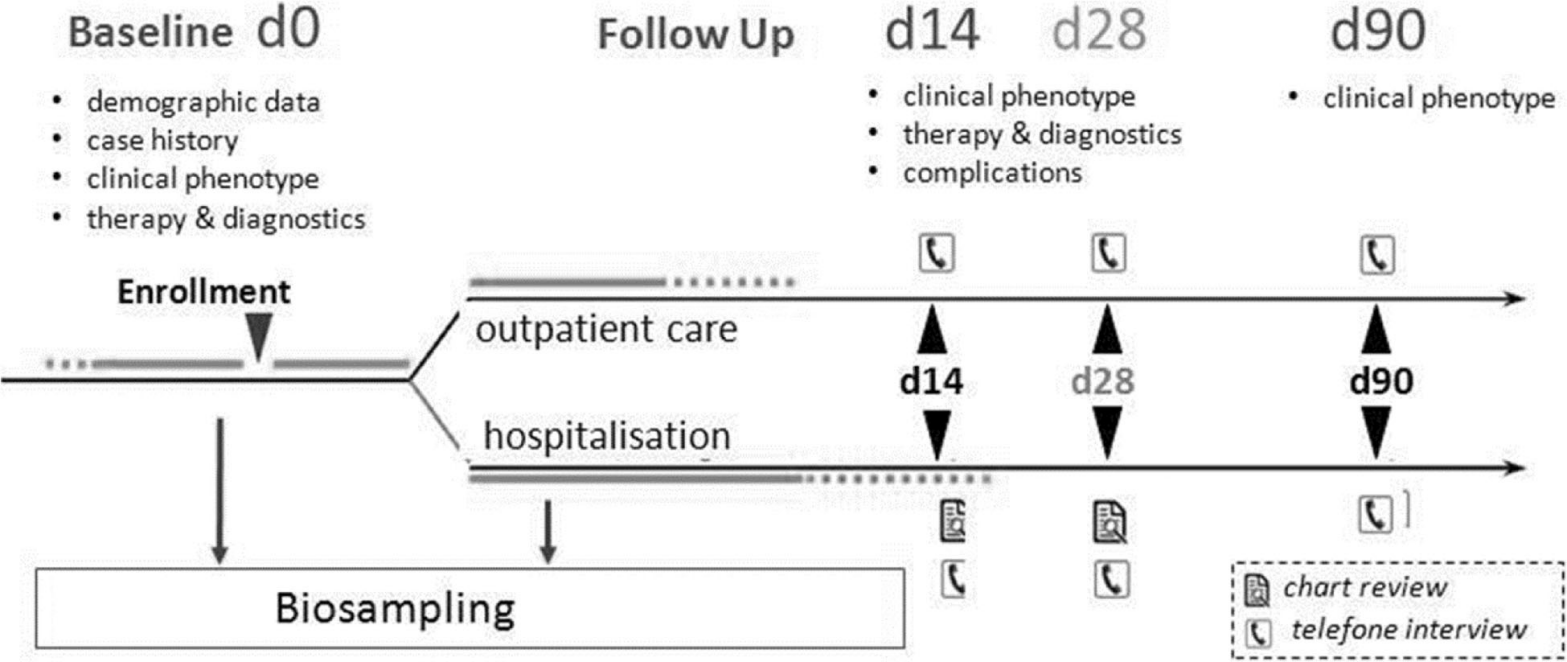
pedCAPNETZ study protocol

pedCAPNETZ is designed as a multicentric, prospective, observational clinical study hosted at Hannover Medical School, Germany. The recruiting network contributing to the registry is currently growing and will consist of clinical centers including pediatric specialists in private practice, outpatient clinics and hospitals at all levels of pediatric health care provision. Detailed data on demographic background, case history, clinical presentation, quality of life, physical examination, diagnostic findings, treatment, socioeconomic measures and other patient related items are collected by means of an electronical case report form (eCRF, Table 1). Over a period of three months upon enrollment, patients are followed, and three follow up visits will be performed (day 14, day 28 and day 90, Fig. 1). During each follow up, data on disease course, treatment, complications, and socioeconomic measures is collected in telephone interviews or, in hospitalized patients, by chart reviews.

**Table 1:** Patient information and clinical data collected in pedCAPNETZ

Type of data	Collected variables
General information	Patient and center identifier, date of inclusion, demographic data (e.g. date of birth, sex, region of origin, parental ethnicity), weight, height
Case history	Date of admission, preexisting and concomitant diseases, medication, vaccinations, surgical interventions
Clinical data	Vital signs, oxygen saturation breathing ambient air, respiratory signs and symptoms, initiated treatment, follow up treatment, complications, symptoms up to day 90
Diagnostics	Diff blood count, CRP, Creatinin, Na, Urea, procalcitonin (optional), interleukin-6 (optional), blood gas (optional), pathogen screening
Imaging	chest radiograph or ultrasound, computed tomography (optional)
Other data	Day care attendance, smoke exposure, household members, number of siblings, family history, socioeconomic disease impact

### In- and exclusion criteria

The study enrolls outpatient and hospitalized children with pedCAP between the first month of life and the 18th birthday. The diagnosis of pneumonia is confirmed by ultrasound or chest x-ray in accordance to the WHO guidelines (presence of consolidations, other infiltrates, or pleural effusions (15)) and the presence of signs or symptoms of an acute lower respiratory tract infection (at least one of the following: [1.] cough, [2.] tachypnea (in accordance to the world health organization (WHO) definition), [3.] fever, and [4.] abnormal findings on auscultation). To avoid misclassification, all chest radiographs and ultrasound findings are retrospectively reviewed by two independent certified pediatric radiologists in accordance to the WHO guidelines (presence of consolidations, other infiltrates, or pleural effusions (15)). Written informed consent will be obtained from all parents and caregivers. Exclusion criteria will be hospitalization for any reason within the last 28 days, congenital or acquired immunodeficiency, cytostatic therapy during past 28 days, neutropenia (< 1000/μl) or relevant immunosuppressive treatment, concomitant respiratory disease with impaired mucociliary clearance such as cystic fibrosis, primary ciliary dyskinesia, tracheostomy, or other severe lung disease including pulmonary tuberculosis.

### Ethics and data protection

The study has been approved by local authorities at all study centers (e.g. ethical approval MHH#2356–2014, Hannover Medical School). A secure information technology platform enabling online data implementation based on the eCRF has been erected. All probands receive a study specific patient pseudonym at the site of recruitment, and all data management queries and further analyses are based on this pseudonym. Collected biosamples are identified by a code linked to the proband’s pseudonym. To secure that analyses cannot be linked to proband identities but that DNA and other proband samples and information will be destroyed upon request, the information linking pseudonyms to patient identities will be held by an external data trustee.

### Biosampling and biobanking

In all probands, comprehensive biosampling is conducted including the collection of whole blood, serum, plasma, urine, sputum, and microbiological swabs (Table 2). Upper and lower airway specimens are screened by PCR and cultured to analyze phenotype specific pathogen spectra and resistance profiles (e.g. viral pathogen PCR, current panel is shown in Additional file 1: Table S2). All collected biosamples, if not used for immediate diagnostic purposes, are processed and stored for regular shipment to the central biobank. All steps are performed according to established standard operational procedures which are available to all study sites in written form and trained prior to first patient inclusion. In complicated pedCAP cases, additional biosampling (e.g. pleural fluid) may be conducted.

**Table 2:** Biomaterials and laboratory parameters collected in pedCAPNETZ

Source	Specimen	Direct analysis	Biobank sampling (planned analyses)
Upper airway tract	Nasopharyngeal aspirate or swab	PCR pathogen screen, microbiome analysis, culture (optional)	No
Lower airway tract	Sputum (± inhalation with NaCl 3%)	PCR pathogen screen, microbiome analysis, culture	Yes
Lower airway tract	Deep throat swab (± inhalation with NaCl 3%)	PCR pathogen screen, microbiome analysis, culture	Yes
Urogenital tract	Urine	Antigen screening: legionella, pneumococcus	Yes
Blood	EDTA	Diff blood count	Yes (genomics, epigenomics)
Blood	Serum	CRP, Creatinin, sodium, urea, procalcitonin (optional), interleukin-6 (optional), blood gas (optional), chlamydia and mycoplasma serology (optional)	Yes (proteomics, metabolomics, antibody-screening)
Blood	Plasma		Yes (microRNA)
Lavage	BAL (optional)	Differential cell count, culture	Yes (microRNA, biomarker analyses)
Others	Pleural effusion (optional), nasal secretions (planned)	Lactate, pH, protein, culture	Yes (biomarker analyses)

### Study centers

**Fig. 2:**
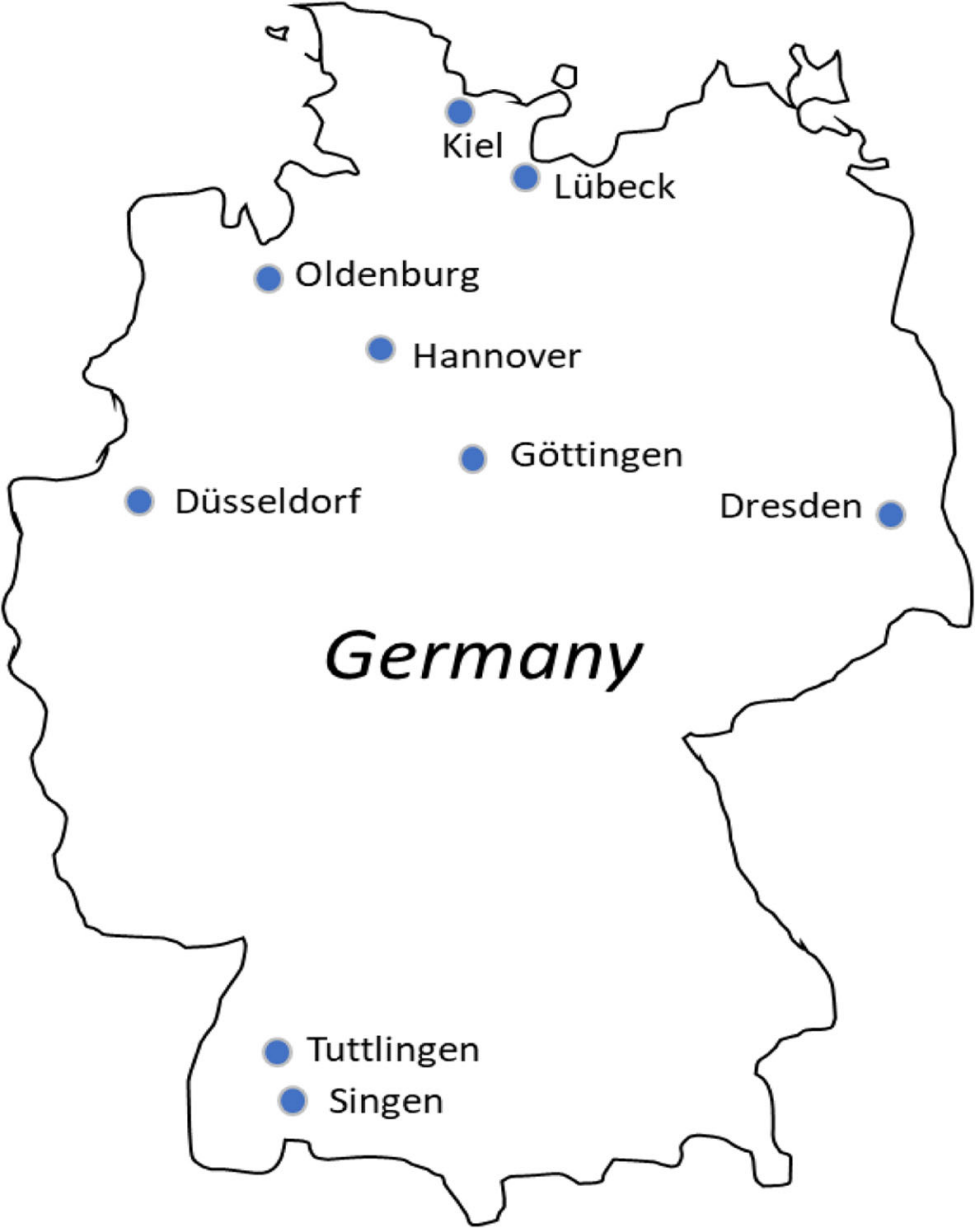
pedCAPNETZ study centers are located in all regions of Germany

Currently, nine local study centers across Germany at Dresden, Düsseldorf, Göttingen, Hannover, Kiel, Lübeck, Oldenburg, Singen, and Tuttlingen have been initiated (Fig. 2). The study sites consist of facilities at all levels of pediatric care ranging from outpatient clinics and private practices to tertiary care hospitals. All study sites undergo extensive training in recruitment, biosample acquisition and processing, as well as in data collection, entry, logistics, and security. pedCAPNETZ will initiate more study cites over Germany in the near future.

### Infrastructure

The pedCAPNETZ registry is embedded into the well-established CAPNETZ infrastructure. Within the first year of pedCAPNETZ funding, all necessary protocols and infrastructure including an online data input platform and a large central pedCAP biobank were developed. The setup includes an online platform for electronic case report form (eCRF) based data input for the collection of thorough epidemiological and clinical data. The central pedCAP biobank enables extensive biosampling for diagnostic purposes and multi-omics analyses. The biobank is located at the Hannover Unified Biobank, (Hannover Medical School, Hannover, Germany). The reference laboratory for pathogen sequencing is located at the of Institute for virology at the University of Freiburg, Germany.

## Discussion

Despite the availability of antibiotics and effective vaccines against most pathogenic bacteria, pedCAP remains a significant healthcare problem not only in countries with low economic standard, but also in the developed world (27). Up until today, pedCAP diagnostics and treatment is only to a small extent evidence based decisions. The pedCAPNETZ initiative aims to improve this situation by delivering thorough clinical and multi–omics data for decision making in pedCAP. Similar to the aims defined during the initial setup of the adult CAPNETZ registry (6), pedCAPNETZ wants to provide the structure to fundamentally improve our knowledge on epidemiology, etiology and management of CAP in children and adolescents.

Since the setup of the registry in 2015, more than *n* = 400 patients were recruited at four study sites, and the network is constantly growing. Thus far, we could achieve follow up rates of 100% on day 14 and 92% on day 90 of the study protocol, with a mean age of patients of 4.2 years (53.2% male patients). Within the next years, we aim at including a total of 1000 study participants.

A central aim of the pedCAPNETZ initiative is the analysis of currently applied diagnostic and therapeutic strategies and their efficacy in pedCAP management. Stringent inclusion criteria and large scale recruitment without preselection for particularly severe or hospitalized cases ensure the collection of updated, valid and comprehensive data on the full spectrum of pedCAP in Germany. This aim is also supported by integrating study partners not only at secondary or tertiary care level, but also from outpatient clinics and primary care practices.

This approach, together with our systemic data and biosampling strategy, allows for validation and development of novel risk stratification scores and biomarkers to identify children at risk for severe disease courses or those that benefit from antibiotic treatment. To this end, we plan to complement the analysis of standard markers of clinical routine with novel diagnostic tools such as high sensitivity screening for serum and nasal fluid inflammatory markers or plasma microRNA screenings. These markers will then be analysed with regard to prediction of disease severity or other pedCAP phenotypic markers. A key question of our future analyses will be how individual pathogen spectra correlate with treatment responses and how the overuse of antibiotics can be limited. We plan to analyse pathogens such as influenza and RSV with state-of-the art methods such as high throughput sequencing in our cases, as these viruses have been demonstrated to be highly prevalent and relevant in pedCAP in other countries (23). Updating etiological data on pedCAP in Germany will have relevant implications in the development of both effective prevention (e.g. vaccination strategies) and management protocols (e.g. rational antibiotic use) in Germany and beyond. pedCAPNETZ aims at contributing to and participating in future decision making processes for guidelines.

To assure the dissemination of the registry, pedCAPNETZ will provide an integrative structure raising clinical and scientific interest and awareness for pedCAP at all levels. An overarching aim of pedCAPNETZ is to build integrative, patient-centred communication structures to connect affected families, primary care givers and clinical centres with diagnostic and research facilities. We will be open to collaborations which can be proposed to the steering committee at any time point. Finally, pedCAPNETZ wants to serve as a platform to perform future interventional studies in pedCAP. Novel diagnostics and treatment approaches can be tested in the framework of the established study network.

To our knowledge, currently no comparable registry exists, neither in Germany nor in Europe. So far, registries focus only on small patient groups with particular phenotypes or pedCAP severity. Moreover, pedCAPNETZ is the first registry to collect information and deeply characterize a large representative sample of children and adolescents with the common disease pedCAP, thus filling an important gap in pediatric and infection health care research. We hope the study results will help to significantly improve the quality of life of pedCAP patients and their families.

## Supplementary information

**Additional file 1.** BMC Pulm Med pedCAPNETZ_Wetzke_Supplementary Material. In this file, two additional tables provide information on (1) current guidelines on antibiotic therapy with references and (2) PCR based screening for CAP associated pathogens

## Data Availability

Data and biomaterials from the pedCAPNETZ can be requested by a formal application and will be decided upon by the board of the study group. Requests should be directed to the corresponding author Gesine Hansen (Hansen.gesine@mh-hannover.de).

## Abbreviations

CAP: Community acquired pneumonia
CAPNETZ: German competence network for CAP
CRP: C-reactive protein
eCRF: Electronical case report form
PCR: Polymerase chain reaction
pedCAP: Pediatric community acquired pneumonia
RSV: Respiratory syncytial virus
RV: Rhinovirus
WBC: White cell counts
WHO: World health organization
