# Supplementary material for: PedCAPNETZ – prospective observational study on community acquired pneumonia in children and adolescents
Source: BMC Pulm Med. 2019 Dec 9;19:238. doi: 10.1186/s12890-019-1013-5 (PMC6902429; doi:10.1186/s12890-019-1013-5)
Supplement: Supplementary file 1 — Additional file 1. BMC Pulm Med pedCAPNETZ_Wetzke_Supplementary Material. In this file, two additional tables provide information on (1) current guidelines on antibiotic therapy with references and (2) PCR based screening for CAP associated pathogens [file 12890_2019_1013_MOESM1_ESM.docx]

**Supplementary material**

***
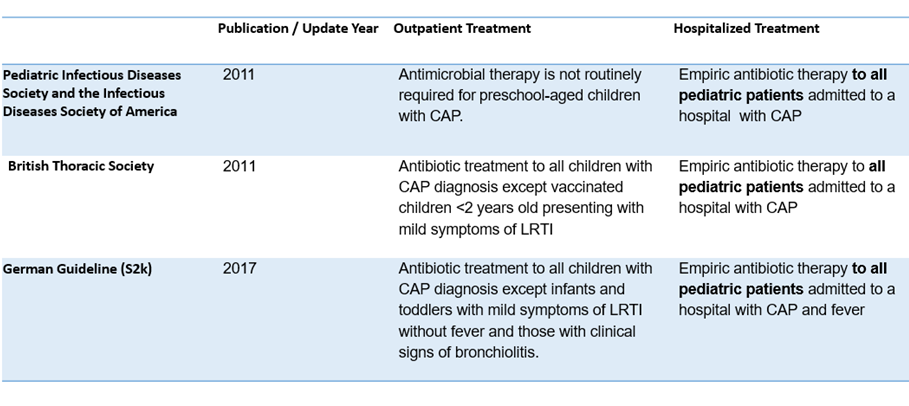
***

Supplementary Table 1: Current pedCAP guidelines: recommendation of antibiotic therapy according to (Supplementary References 1-3)

| **Pathogen screening (PCR)** |
| --- |
| Influenza-A (universal & A/H1N1pdm09) |
| Influenza-B |
| Respiratory syncytial virus A/B |
| Human metapneumovirus A/B |
| Human bocavirus |
| Parainfluenza 1-4 |
| Human corona HKU1,NL63,229,OC43 |
| Rhinovirus |
| Enterovirus |
| Parechovirus |
| Adenovirus |
| Mycoplasma pneumoniae |
| Chlamydia pneumoniae |
| Legionella pneumophilia |
| Bordetella pertussis |

**Supplementary Table 2. PCR based screening for CAP associated pathogens**

**Supplementary References**

1. Bradley JS, Byington CL, Shah SS, Alverson B, Carter ER, Harrison C, et al. Executive summary: the management of community-acquired pneumonia in infants and children older than 3 months of age: clinical practice guidelines by the Pediatric Infectious Diseases Society and the Infectious Diseases Society of America. Clin Infect Dis. 2011;53(7):617-30.

2. AWMF. S2k-Leitlinie „Management der ambulant erworbenen Pneumonie bei Kindern und Jugendlichen (pädiatrische ambulant erworbene Pneumonie, pCAP)“. https://wwwawmforg/uploads/tx_szleitlinien/048-013l_S2k_pCAP_ambulant_erworbene_Pneumonie__Kinder_Jugendliche_2017-06pdf. 2017.

3. Harris M, Clark J, Coote N, Fletcher P, Harnden A, McKean M, et al. British Thoracic Society guidelines for the management of community acquired pneumonia in children: update 2011. Thorax. 2011;66 Suppl 2:ii1-23.
